# The Role of miRNAs in the Prognosis of Triple-Negative Breast Cancer: A Systematic Review and Meta-Analysis

**DOI:** 10.3390/diagnostics13010127

**Published:** 2022-12-30

**Authors:** Talita Araújo B. da S. Santana, Larissa de Oliveira Passamai, Felipe Silva de Miranda, Thaiz Ferraz Borin, Grasiely Faccin Borges, Wilson Barros Luiz, Luciene Cristina Gastalho Campos

**Affiliations:** 1 Department of Health Sciences, State University of Santa Cruz, Ilhéus 45662-900, Bahia, Brazil; 2 Department of Biological Sciences, State University of Santa Cruz, Ilhéus 45662-900, Bahia, Brazil; 3 Laboratory of Tumor Angiogenesis, Georgia Cancer Center, Department of Biochemistry and Molecular Biology, Augusta University, Augusta, GA 30912, USA; 4 Public Policies and Social Technologies Center, Federal University of Southern Bahia, Itabuna 45600-923, Bahia, Brazil

**Keywords:** biomarkers, miRNA, prognosis, TNBC, systematic review, meta-analysis

## Abstract

Breast cancer is one of the most common malignancies among women around the world. The basal or triple-negative subtype (TNBC) is a heterogeneous group of tumors, characterized by its aggressive and metastatic nature, with low survival and worse prognosis. Research on genetic biomarkers, such as microRNAs (miRs) in TNBC, demonstrate their relevance in the prognosis of the disease. Therefore, the objective of this research was to verify the role of miRs in the prognosis of TNBC. A search was carried out in the PubMed (MEDLINE), Web of Science, and Scopus databases, with articles in the English language from 2010 to 2022. Only articles that analyzed the role of miRNAs in the prognosis of TNBC and that met the criteria of the MOOSE method were included. For the preparation and planning of this systematic review, a PRISMA checklist and the MOOSE method were used. The Newcastle–Ottawa Scale was used to analyze the quality of the included studies. The excluded criteria considered were: (1) studies that presented duplication in the databases; (2) reviews of the literature, clinical case reports, meta-analyses, conference abstracts, letters to the editor, theses, dissertations, and book chapters; (3) studies that stratified only women diagnosed with other subtypes of breast cancer subtypes; (4) experiments without a control or comparison group. After the bibliographic survey of the 2.274 articles found, 43 articles met the inclusion criteria, totaling 5421 patients with TNBC analyzed for this review. Six miRs (miR-155, miR-21, miR-27a/b/, miR-374a/b, miR-30a/c/e, and miR-301a) were included in the meta-analysis. A low expression of miR-155 was associated with reduced overall survival (OS) (HR: 0.68, 95% CI: 0.58–0.81). A high expression of miR-21 was a predictor of OS reduction (HR: 2.56; 95% CI: 1.49–4.40). In addition, high levels of miR-27a/b and miR-301a/b were associated with lower OS, while the decreased expression levels of miR-30 and miR-374a/b were associated with worse relapse-free survival (RFS) and shorter disease-free survival (DFS), respectively. The present study revealed that miRs play essential roles in the development of metastases, in addition to acting as suppressors of the disease, thus improving the prognosis of TNBC. However, the clinical application of these findings has not yet been investigated.

## 1. Introduction

Breast cancer (BC) is one of the most common malignancies among women worldwide [1,2]. According to World Health Organization statistics in 2020, 2.3 million women were diagnosed with BC, with a total of 685,000 deaths worldwide. At the end of 2020, 7.8 million women had a diagnosis with the disease in the last 5 years. BC is the most prevalent type in the world [3]. The complex and heterogeneous BC complex, a heterogeneous disease that comprises multiple tumor groups, is associated with different histological patterns, clinical behaviors, and biological characteristics [4]. Triple-negative breast cancer (TNBC) stands out as a highly diverse group of cancers characterized by its aggressive metastatic nature, with poor survival and poor prognosis, representing 15% to 20% of all invasive breast cancers [5,6] and is associated with younger age as a risk factor for developing this subtype [7]. There are several risk factors for developing breast cancer, such as sex, advanced age at menarche [8], family history, reproductive history, number of children, breastfeeding time [9], genetic characteristics [10], race, ethnicity [11], dense breast tissue, certain benign breast conditions, previous chest radiations, radiation therapy [8], anthropometric factors, overweight or obesity [12], menopausal age, menopausal hormone therapy [13], alcohol intake [10], smoking, and physical inactivity [14].

TNBC tumors have larger size, nuclear grade, and mitotic activity, generally have lymph node involvement, and are biologically more aggressive [15]. Studies have extensively documented the clinicopathological risk factors of the disease, such as the status of nodules, tumor size, grade, and the rate of monoclonal antibody marker of cell proliferation (ki-67) [16,17]. However, there are no adequate prognostic and predictive biomarkers for use in clinical practice in TNBC [18]. Research has advanced in discovering possible genetic biomarkers that can contribute to the diagnosis, prognosis, and treatment of the disease [19,20]. Currently, these studies involve the search for microRNAs (miRs) that have scientific relevance in their use as molecular biomarkers. miRs are a new class of endogenous, short-molecule, nonprotein-coding, single-stranded RNAs that are 19 to 25 nucleotides in length. Its function is is to modulate the gene expression in eukaryotes by post-transcriptional regulation. These miRs can act as post-transcriptional silencers, inhibiting the translation of target messenger RNAs [21,22,23,24]. With the advancement of research, there is much evidence that indicates the abnormal expression of miRs may be linked to the development and evolution of cancer [25]. Several of these studies have shown that miRs play significant roles in the prognosis of TNBC [26,27,28,29].

Despite the extensive original publications depicting the different types of miRs and their possible roles as oncogenes and tumor suppressors, it is necessary to organize these published studies and analyze their results. However, to date, only one study has been carried out using a systematic review and meta-analysis, with 19 articles up to the year 2016 [25]. From 2017 to 2022, there was a significant increase in studies that evaluated the role of miRs in the survival of patients with TNBC, and the relevance of the results needs to be analyzed together. In this sense, this work aims to conduct a meta-analysis of studies on the expression of miRs in the prognosis of TNBC, providing a better understanding of the associations between specific miRs.

## 2. Materials and Methods

### 2.1. Protocol and Registration

The present review followed the Preferred Reporting Items for Systematic Review and Meta-Analysis (PRISMA) [30] and the statement and guidelines of the Meta-analysis of Observational Studies in Epidemiology (MOOSE) [31] to conduct this study. The protocol of this systematic review was submitted for registration in the International Prospective Register of Systematic Reviews (PROSPERO) under the registration number CRD42021265841.

### 2.2. Eligibility Criteria

Only original articles that investigated the role of miRs in the prognosis of triple-negative breast cancer were selected. The other inclusion criteria were: (1) original studies, with a date of publication between January 2010 and April 2022, and (2) published in English. The other inclusion criteria that followed the checklist proposed by the MOOSE group were: study design defined as cohort or case–control; described study population (country); sample size (N ≥ 30); description of disease-free survival (DFS) or overall survival (OS) or distant metastasis-free survival (DMFS) or relapse-free survival (RFS) results; described miR measurement method; survival data obtained from a database such asTCGA, PROGmiR, METABRIC, or BreastMark dataset for just external validation the definition of the cut-off values used; the expression of miR measured in cancer samples (tissues) or blood (serum/Plasma); and long follow-up duration (≥60 months). Exclusion criteria were: (1) studies showing duplicate databases; (2) reviews of the literature, editorial notes, posters, clinical case reports, meta-analyses, conference abstracts, letters to the editor, theses, dissertations, and book chapters; (3) studies that stratified only women diagnosed with other subtypes of breast cancer subtypes; and (4) experiments without a control group or comparison group.

### 2.3. Information Sources and Search Strategy

Searches were conducted on 13 April 2022, using electronic databases. PubMed, Scopus, and Web of Science. The search terms in the PubMed database were: “microRNA OR miRNA OR miR” AND “triple-negative breast cancer” AND “prognosis OR prognostic OR survival OR outcome OR mortality”. In the web of science and Scopus, the search was divided into five stages: (1) “microRNA” AND “triple-negative breast cancer” AND “prognosis”; (2) “miRNA” AND “triple-negative breast cancer” AND “prognostic”; (3) “miR” AND “triple-negative breast cancer” AND “outcome”; (4) “microRNA” AND “triple-negative breast cancer” AND “ mortality”; (5) “microRNA” AND “triple-negative breast cancer” AND “survival”.

### 2.4. Study Selection and Data Collection

After completing the searches, a thorough check was carried out on the reference list of the selected articles, to certify that no publication was overlooked. Reference management was performed, and duplicate studies were removed. Studies were selected in two phases. In the first, articles were independently evaluated by two reviewers (T.A.B.S.S. and L.O.P.). They reviewed the titles and abstracts of all references found separately. In the second phase, they read the selected complete texts. Existing conflicts of understanding regarding the articles between the researchers in phases 1 and 2 were resolved by discussion and mutual consent. A third reviewer (T.F.B.) was used when the two reviewers did not reach a consensus or when discrepancies emerged. The reviewers extracted relevant and necessary information from all selected articles, namely the authorship, year of publication, country, study description, study period, duration of follow-up, number of participants and controls, number of cases, type of sample, miRNA analyzed, miRNA expression analysis technique, the expression for worse prognosis, cut-off values, *p*-value, the source of adjusted or unadjusted HR, 95% CI and adjustment for covariates, survival analysis, results, and main conclusions.

### 2.5. Risk of Bias in Individual Studies and Quality Assessment

Methodological quality was independently examined by two reviewers (T.A.B.S.S. and L.O.P.) using the Newcastle–Ottawa Scale (NOS) to reduce the risk of bias. This scale uses a star system (0 to 9) to evaluate selected studies in three domains: selection, comparability, and results. Higher scores represent better quality [32]. Discrepancies were resolved by a third reviewer (T.B.F.). NOS scores varied by study score. In studies in which findings were consistent with strong evidence, they were scored between 6 and 9 and were, therefore, considered of high quality.

### 2.6. Statistical Analysis

Analyses were performed using the Review Manager 5.2 statistical software. The size of the effect between the subgroups in this survival analysis was estimated by the hazard ratio (HR), interpreted as the relative risk of the event occurring as a function of time. HR and 95% CI were determined by inverse variance. The *p*-value was established at 0.05 and the significance level at *p* < 0.001. HR and the 95% confidence interval (95% CI) were manually estimated in studies for which values were not reported. The significance of the combined HR was calculated using the chi-squared test and the magnitude of heterogeneity (I^2^). Heterogeneity was investigated with calculated I^2^ values. When I^2^ was > 50% the statistical heterogeneity was considered substantial [33]. Therefore, for the statistical analysis of the effect of the study concerning heterogeneity (with or without), the random-effect model was applied to calculate the combined HR and meta-regression that was later used to explore the sources of heterogeneity, allowing us to evaluate the effect of multiple factors. Otherwise, when studies were homogeneous, the fixed-effect model was applied. The random- or fixed-effect model by Borenstein et al. [34] was used where the overall effect size (Z) and the *p*-value represented the study group. Additionally, to define the appropriate model, the Review Manager calculator was used to estimate it using the *p*-value and IC of fixed or random effect, resulting in the logarithm (log) of HR. Results were presented through the figure represented by forest and funnel plots.

## 3. Results

### 3.1. Selection of the Study

**Figure 1:**
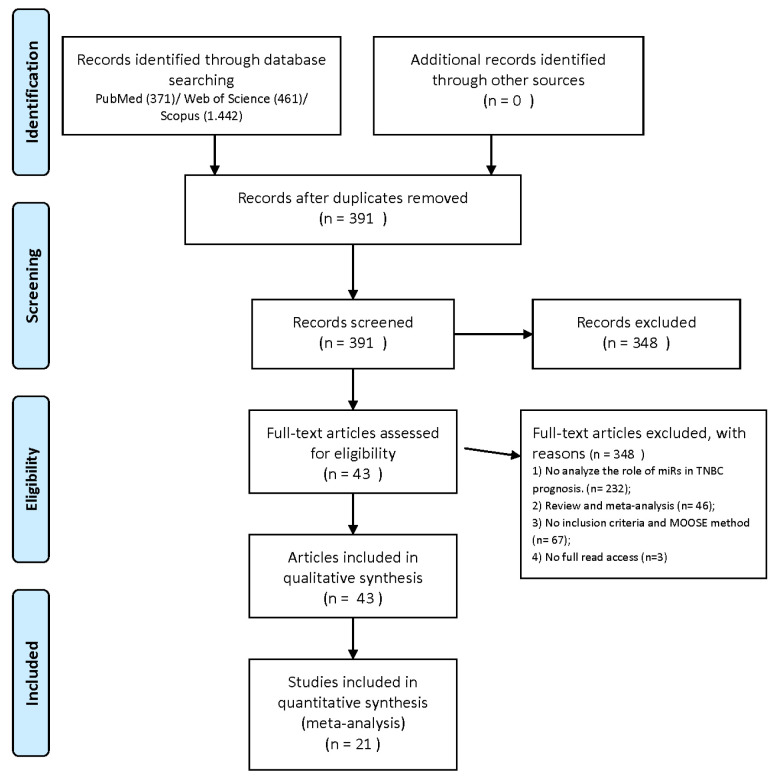
Flowchart of the study selection process.

Figure 1 shows the flowchart for study selection. In the initial stage, 2.274 articles were found in three different databases—PubMed (371), Web of Science (461), and Scopus (1.442). After removing all duplicate studies, 391 articles remained. Subsequently, an evaluation of the abstracts was performed, and articles that did not meet the criteria were excluded, resulting in 43 articles at the end of this stage. No spare articles were identified in the reference lists of studies. Therefore, 43 articles were included in the systematic review [10,28,29,35,36,37,38,39,40,41,42,43,44,45,46,47,48,49,50,51,52,53,54,55,56,57,58,59,60,61,62,63,64,65,66,67,68,69,70,71,72,73,74] and meta-analysis of 21 eligible studies.

### 3.2. Characteristics of the Included Studies

The studies were published from 2011 to 2021, and all were written in English. The characteristics of each study are summarized in Supplementary Table S1. The surveys were carried out in 15 different countries. In 42 articles, they obtained scores ≥ 6, which are considered high scores with strong evidence and consistent findings, and a study by Wang L et al. [64] scored 5 with low quality. Only the study by Uva et al. [62] reached the maximum score. A detail for the notes of each study can be found in Supplementary Table S2. A total of 5421 women over 18 years of age with TNBC were evaluated in the 43 included articles, and the mean follow-up time was 155 months (60–250 months), with an average sample size of 121 patients (39 to 525 patients) in each study.

### 3.3. miRNA Research in the Studies

These studies reported the prognostic values of 61 different miRs. The expression levels of miRs were detected mainly through tumor tissue samples. Additionally, only in four studies were blood samples (serum or plasma) used. During the analysis of the included articles, it was observed that the increase and decrease in the expression of specific miRs, listed in Supplementary Table S2, contributed to a poor prognosis in TNBC and, consequently, to lower survival. Among these miRs, 14 (miR-155, miR-21, miR-27a/b/, miR-374a/b/5p, miR-210, miR-30a/c/e, miR-34a/b/c, miR-454, miR-146a, miR-128, miR-301a, miR-16, miR-493, and miR-181a) were reported by at least two studies, and only miR-155, miR-21, miR-374a/b/5p, miR-30a/c/e, miR-301a/b, and miR-27a/b/ were searched in three or more studies; for this reason, they were included in the meta-analysis.

### 3.4. miR-155 and Prognosis in TNBC

**Figure 2:**
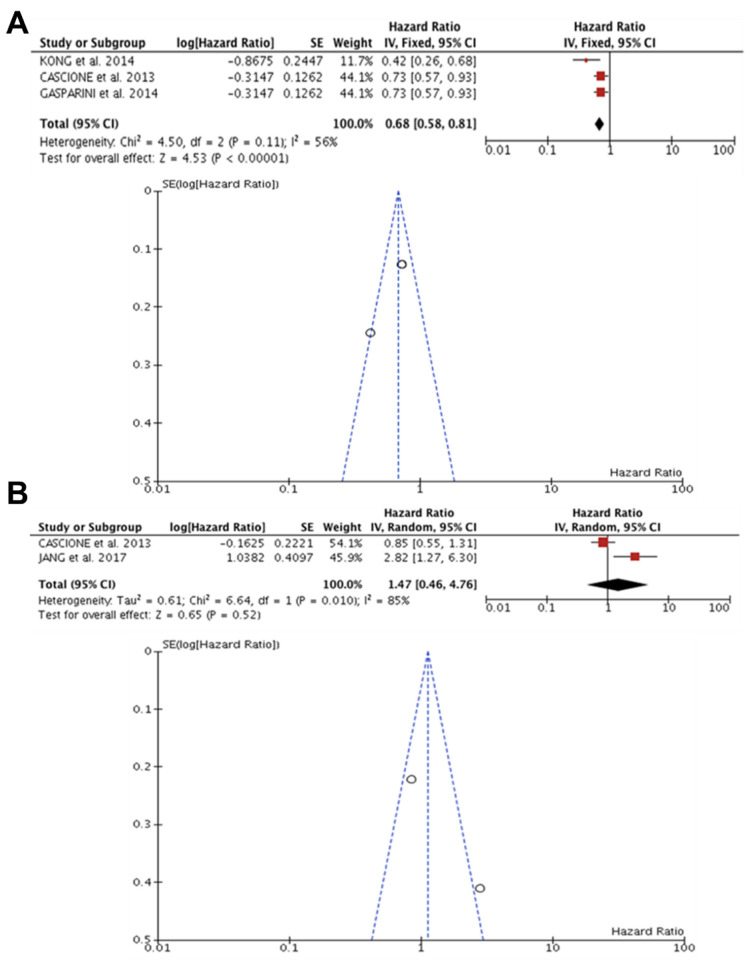
miR-155 and prognosis in TNBC. (**A**) Forest and funnel plot demonstrated miR-155 expression was associated with OS in patients with TNBC (HR: 0.68, 95% CI: 0.58–0.81, I^2^ = 56%, *p* = 0.11), Z = 4, 53 (*p* < 0.00001) [36,42,47]. (**B**) Forest and funnel plot showed miR-155 expression was not associated with DFS in patients with TNBC and in addition to significant heterogeneity (HR: 1.47; 95% CI: 0.46–4.76; I^2^ = 85%, *p* = 0.010) [36,44].

The meta-analysis included four articles [36,42,44,47] (n = 767). In the studies by Gasparini et al. [42], Kong et al. [47], and Cascione et al. [36], the analysis observed substantial heterogeneity (I^2^ = 56%, *p* = 0.11) and also revealed that the expression of miR-155 was associated with OS in patients with TNBC (HR: 0.68, 95% CI: 0.58–0.81), Z = 4.53 (*p* < 0.00001) (Figure 2A). Regarding DFS, studies by Cascione et al. [36] and Jang et al. [44] were found to have significant heterogeneity (I^2^ = 85%, *p* = 0.010), in addition to the expression of miR-155 not being associated with DFS in patients with TNBC (HR: 1.47; 95% CI: 0.46–4.76) (Figure 2B).

### 3.5. miR-21 and Prognosis in TNBC

**Figure 3:**
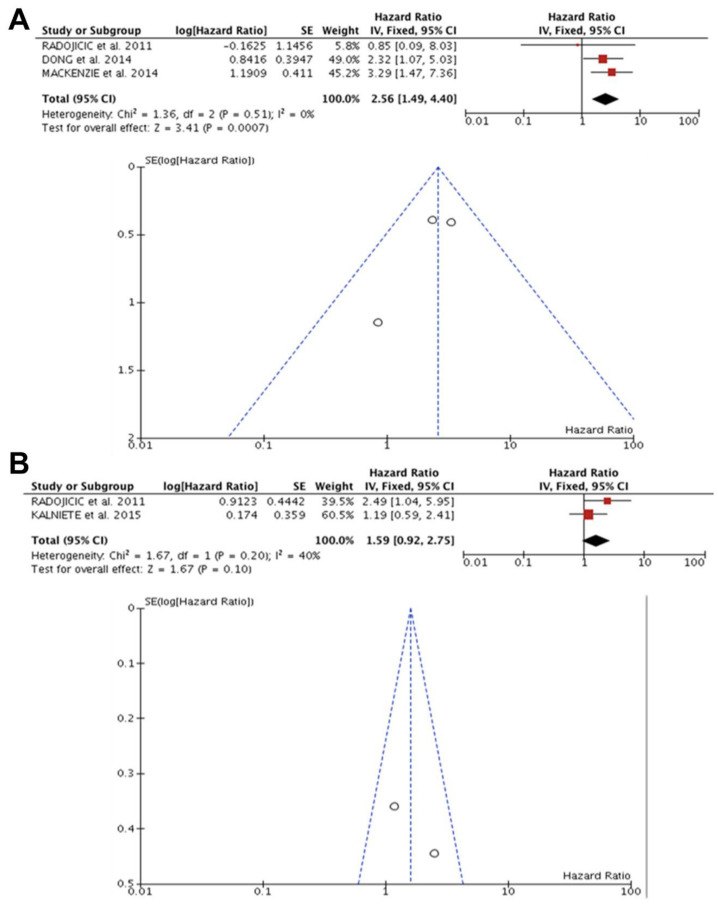
miR-21 and prognosis in TNBC. (**A**) Forest and funnel plot showed that high miR-21 expression was related to worse OS prognosis in patients with TNBC (HR: 2.56; 95%CI: 1.49–4.40; I^2^ = 0.0%, *p* = 0.51) [41,53,54]. (**B**) Forest and funnel plot presented DFS in patients with TNBC was not related to a worse prognosis. Homogeneity was observed, but the effect was not significant (HR: 1.59; 95% CI: 0.92–2.75; I^2^ = 40%, *p* = 0.20) [45,54].

All four articles (n = 276) reported the effect of a high miR-21 expression on a worse prognosis of patients with TNBC. Of these studies, only Radojicic et al. [54] described the OS and DFS data, Mackenzie et al. [53] and Dong et al. [41] reported only the OS data, and Kalniete et al. [45] referred only to the DFS data. No heterogeneity was observed between the studies (OS, I^2^ = 0.0%, *p* = 0.51; DFS, I^2^ = 40%, *p* = 0.20). The effects of miR-21 expression on OS were significant (HR: 2.56; 95%CI: 1.49–4.40). However, in DFS, despite homogeneity between studies, the effect was not significant (HR: 1.59; 95% CI: 0.92–2.75) on the association of prognosis in patients with TNBC (Figure 3A,B).

### 3.6. miR-30 and the Prognosis in TNBC

**Figure 4:**
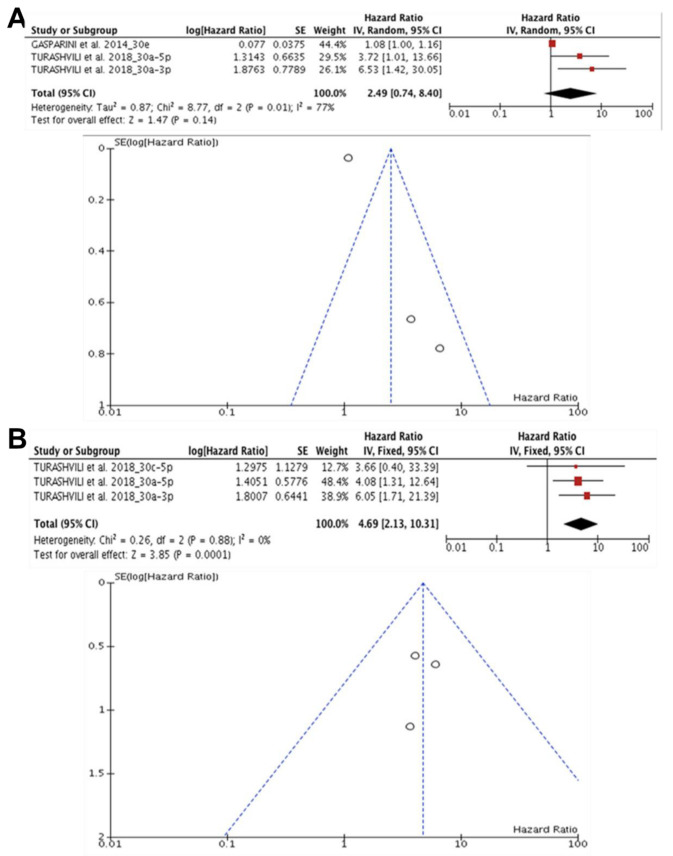
miR-30 and the prognosis in TNBC. (**A**) Forest and funnel plot related low miR-30 expression (30e, 30a-5p, 30a-3p, 30c-5p) is associated with poor prognosis in OS in patients with TNBC, (HR: 2.49, 95% CI: 0.79–8.40; I^2^ = 77%, *p* = 0.01, Z = 1.47 (*p* = 0.14) [42,62].(**B**) Forest and funnel plot demonstrated that miR-30 expression significantly interfered with the RFS of patients with TNBC (HR: 4.69, 95% CI: 2.13–10.31; I^2^ = 0.0%, *p* = 0.88)), Z = 3.85, (*p* < 0.0001) [62].

Four studies (n = 224) suggested that the downregulation of miR-30 (30e, 30a-5p, 30a-3p, 30c-5p) is associated with a poor prognosis in patients with TNBC. The study by Gasparini et al. [42] only obtained data from the OS. In Turashvili et al. [62], analyses were performed with the OS and RFS (relapse-free survival) data. In the results of the studies related to OS, significant heterogeneity was observed (I^2^ = 77%, *p* = 0.01), and the effect of specific miR expression was not significant on the patient’s prognosis (HR: 2.49, 95% CI: 0.79–8.40), Z = 1.47 (*p* = 0.14) (Figure 4A). The RFS data did not reveal heterogeneity (I^2^ = 0.0%, *p* = 0.88) between studies and demonstrated that miR-30 expression significantly interfered with the RFS of patients with TNBC (HR: 4.69, 95% CI: 2.13–10.31), Z = 3.85, (*p* < 0.0001) (Figure 4B).

### 3.7. miR-374 and Prognosis in TNBC

**Figure 5:**
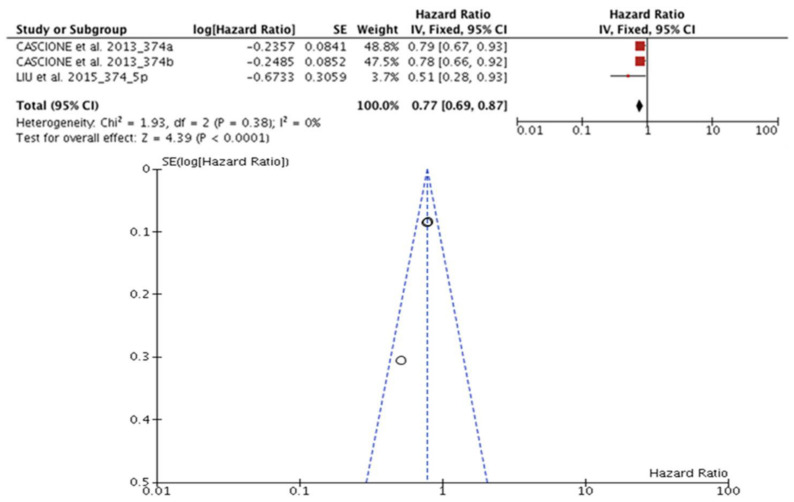
miR-374 and prognosis in TNBC. Forest and funnel plot showed effect of miR-374 downregulation was associated with a shorter DFS (HR: 0.77; 95% CI: 0.69–0.87; I^2^ = 0%, *p* = 0.38), Z = 4.39 (*p* < 0.0001) [36,51].

Two articles (n = 629) evaluated the association of miR-374a/b expression with prognosis in TNBC. Liu Y. et al. [51] reported OS and DFS data, and Cascione et al. [36] reported only the DFS data. Therefore, only DFS statistical calculations were analyzed. All of these studies provided the HR data adjusted for DFS, and no heterogeneity was observed (I^2^ = 0%, *p* = 0.38). Therefore, the effect of miR-374 downregulation was associated with a shorter DFS (HR: 0.77; 95% CI: 0.69–0.87), Z = 4.39 (*p* < 0.0001) (Figure 5).

### 3.8. miR-301 and Prognosis in TNBC

**Figure 6:**
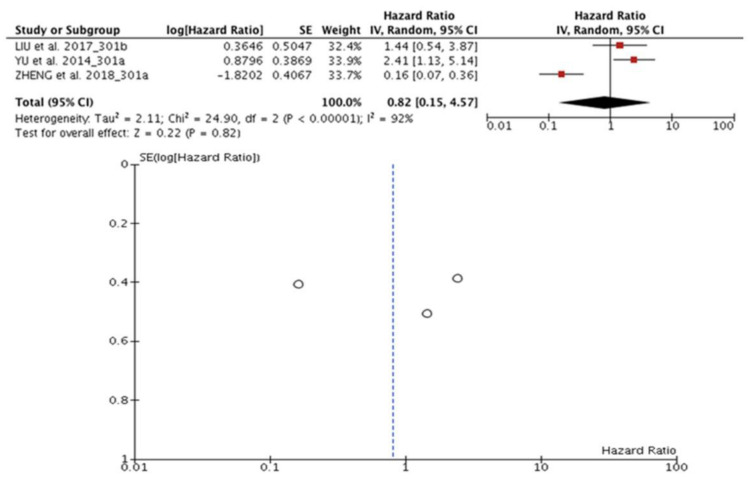
miR-301 and prognosis in TNBC. Forest and funnel plot observed effect of miR-301 a/b expression on OS in TNBC was not significant (HR: 0.82; 95% CI: 0.15–4.57; I^2^ = 92%, *p* < 0.00001), Z = 0, 22, (*p* = 0.82) [29,68,74].

Three articles (n = 322) evaluated the association between miR-301a/b expression and prognosis in TNBC. Li HY et al. [29] and Yu et al. [68] provided only OS data, and in the study of Zheng et al. [74], the OS and DFS data were made available. The HR data in one study were extracted through the survival curve; however, the other two HRs were adjusted. Among these studies, significant heterogeneity was observed (I^2^ = 92%, *p* < 0.00001). Therefore, due to the set of studies, the effect of miR-301 a/b expression concerning OS in TNBC (HR:0.82; 95% CI: 0.15–4.57), Z = 0, 22, (*p* = 0.82) was not expressive, consequently without a significant result (Figure 6).

### 3.9. miR-27 and Prognosis in TNBC

**Figure 7:**
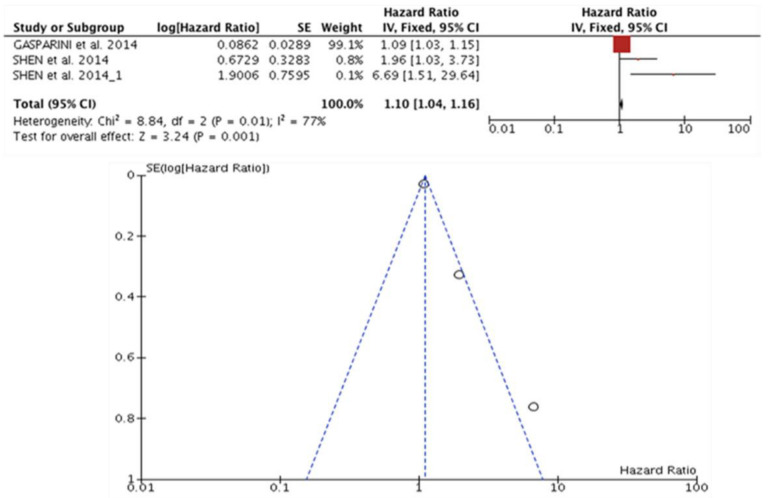
miR-27 and prognosis in TNBC. High miR-27a/b expression in TNBC patient is associated with worse prognosis in OS (HR: 1.10; 95% CI: 1.04–1.16; I^2^ = 77%, *p* = 0.01), Z = 3.24 (*p* < 0.001) [42,57].

Four studies (n = 920) evaluated the association between a high expression of miR-27a/b and a worse prognosis in TNBC. Gasparini et al. [42] and Shen et al. [57] provided the OS data. Of these three articles, only Liu Y. et al. (2015) provided the DFS data. Univariate HR was calculated in the study by Gasparini et al. [42] for OS, and two studies of multivariate analyses were performed. Among these studies, significant heterogeneity was observed (I^2^ = 77%, *p* = 0.01). Regarding the expression of miR-27 a/b and OS in TNBC, it was significant at 1.10 (95% CI: 1.04–1.16), Z = 3.24 (*p* < 0.001) (Figure 7).

## 4. Discussion

Breast cancer is a heterogeneous disease with different molecular subtypes. TNBC is the second most common subtype of BC in women worldwide [75], accounting for 15–20% of all BC. In recent years, many reports have shown differential expression levels of miRs in BC [76,77]. However, in general, these studies evaluated specific miRs or combinations of multiple miRs for the diagnosis of BC, and the miR expression for each molecular subtype of CM remains poorly explored. Specifically, the TNBC subtype shows distinct histopathological characteristics and clinical behavior featuring an aggressive phenotype [78]. This subtype usually has early and high recurrence rates, resulting in poor survival, as well as the patients not receiving treatment-directed therapy [79]. The absence of targeted therapy is one of the obstacles in the treatment of TNBC, which may partially explain the worse prognosis in patients with TNBC than those with other subtypes of BC [5]. In this context, it becomes interesting to search for specific miRs for the diagnosis of TNBC to direct the treatment and improve the clinical outcome of this disease.

Therefore, this systematic review and meta-analysis aimed to verify the role of miRs as predictive biomarkers that can be used in the prognosis of TNBC, thus comparing the effect of a specific miR among the study population. This work provide an up-to-date review of the topic with 43 articles between cohort and case–control studies that examined the role of miRs in the prognosis of TNBC. The pattern of several miRs that appear to be altered in TNBC can be observed, suggesting the possibility of being used as a prognostic tool in the disease [41,56]. For example, the reduced expression of miR-155 predicted poor overall survival in patients with TNBC, while the elevated levels of miR-21, miR-27a/b, miR-210, and miR-454 were associated with shorter overall survival [19,55,80]. Similarly, a decrease in the miR-374a/b/5p expression and an increase in miR-454 levels were correlated with lower disease-free survival [28,36]. Other miRs have been associated with chemoresistance during treatment [42,56,57].

miR-155 appears to be a promising prognostic tool in many types of cancer, especially BC. Generally, miR-155 is considered an oncogenic miR that promotes tumor growth, angiogenesis, and BC aggressiveness [81]. However, in patients with TNBC, this miR has the opposite behavior; that is, the overexpression of miR-155 has a protective effect, increasing survival. This may be due to the various molecular mechanisms found in TNBC that may play a role in DNA damage pathways [42,44]. miR-21 was one of the first oncomiRs identified as overexpressed in breast carcinoma. Its function has been correlated with cell survival, proliferation, stimulating invasion, extravasation, and metastases [82], which has been associated with advanced clinical stage, lymph node metastasis, and poor prognosis of the patient [83]. The target of miR-21 may be pro-apoptotic phosphatase and the tensin homolog (PTEN), which promote tumor cell proliferation by inhibiting tumor cell apoptosis [41]. miR-27a/b is one of the significantly increased miRs in TNBC, and this high expression has been associated with poor overall patient survival [42]. miR-27a is an oncogenic microRNA, and its elevated expression is associated with poor overall survival in BC patients, which suggests that miR-27a may be an important biomarker in disease progression; high levels of miR-27a appear to be significantly correlated with tumor size, lymph node metastases, distant metastases, and poor prognosis in patients with BC [60]. The peroxisome proliferator-activated receptor (PPAR) and PTEN signaling in TNBC cells act as tumor suppressors, regulating the cell division cycle gene (CDC27), which is an essential component of the anaphase promoter complex (APC) important in the regulation of the mitotic checkpoint to ensure chromosomal integrity [50,84]. miR-374a has been reported as a “protective” gene in invasive breast cancer. In analyzing the mRNA profile of this miR, researchers observed that an important tumor suppressor inhibitor of cyclin-dependent kinase 2A (CDKN2A) is also a target of miR-374a. Furthermore, it has been associated with better survival in lung cancer due to this protective ability [36]. The in vitro study by Liu Y. et al. [51] showed that miR-374b-5p overexpression reduced tumor cell invasion, supporting the epidemiological findings that it is associated with better outcomes for TNBC [51]. However, a study by Son et al. [85] with in vitro and in vivo models indicated that a high expression of miR-374a-5p promotes tumor progression in TNBC. Therefore, miR-374a-5p may be a potential prognostic marker of TNBC [85].

MiR-30a expression was significantly reduced in TNBC, and patients with a low miR-30a expression were associated with a higher histological grade and more lymph node metastases. Furthermore, researchers found that miR-30a curtailed the epithelial-mesenchymal transition (EMT) of TNBC cells. The overexpression of miR-30a increased the expression of the epithelial marker E-cadherin but decreased the expression of the mesenchymal markers N-cadherin and vimentin. In this study, it was shown that the overexpression of miR-30a significantly suppressed the invasion and migration of cancer cells [86]. A reduced expression of miR-34a/34b/34c is strongly associated with tumor progression and a worse prognosis. Furthermore, miR-34c was an independent risk factor for OS in patients with TNBC [73]. However, in a study with 39 patients, it was observed that high levels of miR-34b expression negatively correlated with DFS and OS of patients with TNBC [26]. The high expression of miR-301a was responsible for cell proliferation induced by the cancerous protein phosphatase 2A inhibitor (Cip2a) and cell invasion. MiR-301a levels were overexpressed in tumor tissues compared with adjacent tissues. Furthermore, the level of miR-301a in TNBC cells increased compared with other subtypes [87]. miR-301a level was positively correlated with size, depth of invasion, stage of TNM, and lymph node metastasis. The expression of MiR-301a was an independent prognostic factor for survival in patients with TNBC [68].

miR-210 is highly expressed in TNBC, and its overexpression has been associated with a poor prognosis of the disease [88]. Consequently, a low expression of miR-210 has been shown to improve DFS and OS more than a high expression [89]. The miR-210 signal was detected in tumor cells, and in the tumor microenvironment, in a positive region for the pan-leukocyte marker—the common leukocyte antigen (CD45-LCA). miR-210 is hypoxia-regulated and is a target of hypoxia-inducible factor 1 alpha (HIF1-alpha). As hypoxia is an important feature of solid tumors, miR-210 has been shown to be overexpressed in several types of cancer, including breast, lung, head, and neck, pancreatic cancer, or glioblastoma [88]. In addition to being correlated with lymph node metastases, clinical staging, and differentiation, it may act as a promising prognostic tool for breast cancer [89]. miR-454 promoted the proliferation of TNBC and collaborated in the migration and invasion of TNBC cells. Furthermore, it improved the survival of TNBC cells after ionizing radiation. miR-454 inhibited radiation-induced apoptosis in TNBC cells by regulating the expression of caspase 3/7 and the antiapoptotic protein BCL-2. The levels of phosphatase tensin homolog (PTEN) and pAKT in TNBC cells were found to change after the overexpression of miR-454 [90]. A case–control study found that miR-454 upregulated TNBC tissues and cell lines. Therefore, the fall in miR-454 inhibited the progression of TNBC by suppressing cell proliferation and EMT and inducing cell apoptosis, indicating a promising treatment strategy for patients with TNBC [91].

The rate of miR-146a in TNBC tissues was significantly higher than its rates in other BC subtypes and is related to larger tumor size and histological stage. In this study, patients with a high miR-146a expression were found to have a lower survival rate than individuals with a low expression. miR-146a is highly expressed in TNBC, leading to a worse prognosis, making miR-146a expression an independent factor that affects the prognosis of patients [66]. However, in another study, the results suggested that the high expression of miR-146a improves overall survival in patients with TNBC and BRCA1 deficiency [72]. miR-128 has been investigated in a series of in vitro and in vivo experiments to investigate the function and mechanism in the development of invasive ductal breast cancer. As a result of this study, we found that a low miR-128 expression was correlated with lower OS and DFS in TNBC, as well as in other subtypes. In addition, miR-128 was able to inhibit glucose metabolism, mitochondrial respiration, and TNBC cell proliferation. These effects were consistent with the direct inhibition of the insulin receptor miR-128 and the insulin receptor substrate 1 [71]. MiR-181 expression in TNBC tissue samples was found to increase compared with nontumor tissues. A high expression of miR-181a has been associated with a higher tumor grade, lymph node metastasis, and chemoresistance [10]. Furthermore, miR-181a expression was significantly correlated with worse OS in TNBC patients with or without chemotherapy [29]. Multivariate analyses revealed that miR181a was an independent prognostic factor of OS in TNBC [10]. The expression level of miR-493 is a significant prognostic factor in breast cancer. During the Kaplan–Meier analysis, one study observed that patients with a high miR-493 expression had better disease-free survival than patients with a low expression, soon after adjusting for clinical pathological factors common in breast cancer. Further subtype analyses revealed that miR-493 expression levels were only significantly seen in patients with TNBC [70]. The decreased expression of miR-16 was associated with the worsening of OS and DMFS in patients with TNBC, as shown by studies by Cascione et al. [36] and De Rinaldis et al. [40]. Therefore, the overexpression of miR-16 decreases cell growth and proliferation, inducing apoptosis in human cancer. However, Usmani et al. [92], using a sample of 194 participants, found a high expression of this miR in all stages of BC, especially in relation to patients with TNBC who showed higher expression levels of this miR, concluding that miR-16 is an important oncogene and has high functionality for prognostic biomarker and disease diagnosis [92].

In summary, the results of this systematic review demonstrated that the elevated levels of miR-21, miR-27a/b, miR-210, miR-181a, miR-301a/b, and miR-454 were associated with a reduction in overall survival time. Likewise, a higher miR-454 expression was associated with shorter DFS. However, the lower expression levels of miR-128, miR-34 a/b/c, miR-30a/c/e, and miR-16 miR-155, miR-374a/b/5p, and miR-493 were associated with the worsening of OS, DMFS, DFS, and RFS in patients with TNBC. In the same sense, statistical analyses corroborate the above results, except for the effect on miR-301a/b and its high expression concerning OS, which did not obtain a significant result. Some limitations can be observed in this work; for example, there are varieties of miRs found in the studies compared with studies that portray the same specific miR. Another example found was the marked heterogeneity in some analyses, which was certainly due to differences in sample size, cohort or case–control demographics, patient characteristics, prognostic factors related to tumor size, grade, differentiation, stage, the lymph node involvement axillary, and high-risk genetic mutations. In addition, various survival assessment models were also used, such as OS, DMFS, DFS, and RFS, the use of different miR detection methods and cut-off values for expression levels, sample types, the methods of preparing these samples, schedule, duration of follow-up, and HR extraction methods. Thus, we suggest that further studies should be conducted to evaluate specific miRs as promising tools in the prognosis of TNBC.

## 5. Conclusions

The present study demonstrated that miRs are the main regulators of gene expression under normal and pathological physiological conditions, playing an essential role in the development, progression, and evolution of metastases. However, they also act as disease suppressors, improving the prognosis of TNBC. We observed that an increased or decreased expression of certain miRs was associated with reduced survival in TNBC. Therefore, studies support the possibility that specific miRs can be used as potential biomarkers to predict response to radiotherapy, chemotherapy, and prognosis in TNBC. However, the clinical application of these findings requires future evaluation with a greater number of studies investigating the prognostic value of miRs.

## Data Availability

Not applicable.

## Supplementary Materials

The following supporting information can be downloaded at: https://www.mdpi.com/article/10.3390/diagnostics13010127/s1, Supplemental File S1: Descriptive characteristics and statistical data of the studies.
