# Supplementary material for: The Role of miRNAs in the Prognosis of Triple-Negative Breast Cancer: A Systematic Review and Meta-Analysis
Source: Diagnostics (Basel). 2022 Dec 30;13(1):127. doi: 10.3390/diagnostics13010127 (PMC9818368; doi:10.3390/diagnostics13010127)
Supplement: Supplementary file 1 [file diagnostics-13-00127-s001.zip › diagnostics-1815610-supplementary.pdf]

Table S1: Descriptive characteristics and statistical data of the studies (n=41)

Author/ Year	miRNA	Country	Study type	N	Assay	Sample	Survival Analysis	Source of HR	Adjusted	Cut-off value	Follow-up time (months)
Cao et al. (2016) [28]	454	China	Cohort	92	ISH	FFPE	OS/DFS	Report	Yes	Score >4	145
Cao et al. (2016) [28]	454	China	Cohort	116	ISH	FFPE	OS/DFS	Report	Yes	Score >4	125
Cao Z. et al. (2016) [35]	361-5p	China	Cohort	111	ISH	FFPE	DFS	Report	Yes	Intensity >2	96,37
Cascione et al. (2013) [36]	16	EUA	Cohort	173	qRT-PCR	BP	OS/DFS	Report	Yes	Risk- score	194
Cascione et al. (2013) [36]	155	EUA	Cohort	173	qRT-PCR	BP	OS/DFS	Report	Yes	Risk- score	194
Cascione et al. (2013) [36]	374[a]	EUA	Cohort	173	qRT-PCR	BP	DFS	Report	Yes	Risk- score	194
Cascione et al. (2013) [36]	125b	EUA	Cohort	173	qRT-PCR	BP	OS/ DFS	Report	Yes	Risk- score	194
Cascione et al. (2013) [36]	374b	EUA	Cohort	173	qRT-PCR	BP	DFS	Report	Yes	Risk- score	194
Cascione et al. (2013) [36]	497	EUA	Cohort	173	qRT-PCR	BP	DFS	Report	Yes	Risk- score	194

Author/ Year	miRNA	Country	Study type	N	Assay	Sample	Survival Analysis	Source of HR	Adjusted	Cut-off value	Follow-up time (months)
Cascione et al. (2013) [36]	655	EUA	Cohort	173	qRT-PCR	BP	DFS	Report	Yes	Risk- score	194
Cascione et al. (2013) [36]	421	EUA	Cohort	173	qRT-PCR	BP	DFS	Report	Yes	Risk- score	194
Chen et al. (2017) [37]	211-5p	China	Case control	82	qRT-PCR	Serum	OS/RFS	Report	No	Risk- score	>100
Chen L. et al. (2021) [38]	223	China	Cohort	150	ISH	FFPE	OS/DFS	Report	Yes	Intensity > 4	96,02
D. Rinaldis et al.(2013)[40]	16-2*	England	Cohort	114	IHC / CISH	Tissue	DMFS	Report	No	Risk- score	66
D. Rinaldis et al.(2013)[40]	766	England	Cohort	114	IHC /CISH	Tissue	DMFS	Report	No	Risk- score	66
D. Rinaldis et al.(2013)[40]	381	England	Cohort	114	IHC /CISH	Tissue	DMFS	Report	No	Risk- score	66
D. Rinaldi et al.(2013)[40]	409-5p	England	Cohort	114	IHC /CISH	Tissue	DMFS	Report	No	Risk- score	66
D. Rinaldis et al.(2013)[40]	17-92	England	Cohort	114	IHC / CISH	Tissue	DMFS	Report	No	Risk- score	66
D. Rinaldis et al.(2013)[40]	106b-25	England	Cohort	114	IHC/ CISH	Tissue	DMFS	Report	No	Risk- score	66

Author/ Year	miRNA	Country	Study type	N	Assay	Sample	Survival Analysis	Source of HR	Adjusted	Cut-off value	Follow-up time (months)
Deng et al (2017) [39]	221-3p	China	Case control	125	qRT-PCR	FFPE	DFS	Report	Yes	Risk- score	1 71
Dong et al. (2014) [41]	21	China	Case control	72	qRT-PCR	Tissue	OS	Report	No	Median	96
Gasparini et al. (2014)[42]	155	EUA	Cohort	173	Tissue Microarray	BP	OS	Report	No	Median	200
Gasparini et al. (2014)[42]]	493	EUA	Cohort	173	Tissue Microarray	BP	OS	Report	No	Median	200
Gasparini et al. (2014)[42]	30e	EUA	Cohort	173	Tissue Microarray	BP	OS	Report	No	Median	200
Gasparini et al. (2014)[42]	27a	EUA	Cohort	173	Tissue Microarray	BP	OS	Report	No	Median	200
Gong et al.2021 [43]	378	Germany	Cohort	103	qRT-PCR	Tissue	OS/DFS	Report	Yes	Median	>70
Jang et al. (2017) [44]	155	Korea	Cohort	190	qRT-PCR	BP	DFS/DMFS	Report	No	Median	150
Jang et al. (2017) [44]	9	Korea	Cohort	190	qRT-PCR	BP	DFS/DMFS	Report	No	Median	150
Kalniete et al. (2015) [45]	214	Latvia	Cohort	50	qRT-PCR	FFPE	DFS	SC	No	Median	120
Kalniete et al. (2015) [45]	21	Latvia	Cohort	50	qRT-PCR	FFPE	DFS	SC	No	Median	120
Kalniete et al. (2015) [45]	10b	Latvia	Cohort	50	qRT-PCR	FFPE	DFS	SC	No	Median	120

Author/ Year	miRNA	Country	Study type	N	Assay	Sample	Survival Analysis	Source of HR	Adjusted	Cut-off value	Follow-up time (months)
Kim et al.(2019) [46]	34a	Korea	Cohort	114	qRT-PCR	Tissue	OS/DFS	Report	Yes	0,90	61
Kong et al. (2014) [47]	155	EUA	Cohort	231	qRT-PCR ISH/HC	frozen tissue/FFPE	OS	Report	Yes	Levels 90	120
Li, HY et al. (2017)[29]	105	Taiwan	Cohort	74	qRT-PCR	Plasma /tissue	OS	SC	No	ROC curve (1,31)	>200
Li, HY et al. (2017)[29]	301b	Taiwan	Cohort	74	qRT-PCR	Plasma /tissue	OS	SC	No	ROC curve (1,31)	>200
Li, HY et al. (2017)[29]	181a	Taiwan	Cohort	74	qRT-PCR	Plasma /tissue	OS	SC	No	ROC curve (1,31)	>200
Li, HY et al. (2017)[29]	93-3p	Taiwan	Cohort	74	qRT-PCR	Plasma /tissue	OS	SC	No	ROC curve (19.23)	>200
Li, Jie et al. (2017)[48]	17-5p	China	Case control	82	qRT-PCR ISH/ IHC	Tissue	OS	Report	Yes	Risk- score	125
Liu J. et al.(2016)[49]	497	China	Case control	47	qRT-PCR	Tissue	OS	SC	No	Median	80
Liu, P. et al. (2015)[50]	26a	China	Case control	41	qRT-PCR	Tissue	OS/DSF	SC	Yes	Median	120
Liu, Y. et al. (2015) [51]	374b-5p	China	Cohort	456	NSCA	Tissue	OS/DFS	Report	Yes	Quartile	107
Liu, Y. et al. (2015) [51]	218-5p	China	Cohort	456	NSCA	Tissue	OS/DFS	Report	Yes	Quartile	107
Liu, Y. et al. (2015) [51]	126-3p	China	Cohort	456	NSCA	Tissue	OS/DFS	Report	Yes	Quartile	107

Author/ Year	miRNA	Country	Study type	N	Assay	Sample	Survival Analysis	Source of HR	Adjusted	Cut-off value	Follow-up time (months)
Liu, Y. et al. (2015) [51]	27b-3p	China	Cohort	456	NSCA	Tissue	OS/DFS	Report	Yes	Quartile	107
Lv, Z. et al. (2017)[52]	212-5p	China	Case control	125	qRT-PCR	Tissue	OS/DSF	Report	Yes	Median	120
Mackenzie et al. (2014)[53]	21	EUA	Cohort	105	ISH/ IHC	Tissue	OS	Report	No	Risk-score	180
Radojicic et al. (2011)[54]	21	Greece	Case control	49	qRT-PCR	FFPE	OS/DSF	SC	No	Median	120
Radojicic et al. (2011)[54]	210	Greece	Case control	49	qRT-PCR	FFPE	OS/DSF	SC	No	Median	120
Romero - C. et al. (2018)[55]	342-3p	Mexico/ Italy	Cohort	132	qRT-PCR	FFPE	OS	SC	No	Quartile	>250
Sahlberg et al. (2015) [56]	18b	Norway	Cohort	70	qRT-PCR	Serum	OS	Report	Yes	Risk-score	>60
Sahlberg et al. (2015) [56]	103	Norway	Cohort	70	qRT-PCR	Serum	OS	Report	Yes	Risk-score	>60
Sahlberg et al. (2015) [56]	107	Norway	Cohort	70	qRT-PCR	Serum	OS	Report	Yes	Risk-score	>60
Sahlberg et al. (2015) [56]	652	Norway	Cohort	70	qRT-PCR	Serum	OS	Report	Yes	Risk-score	>60
Schwarzenbacher et al. (2019)[59]	1287-5p	Italy	Cohort	131	qRT-PCR	Tissue	RFS	Report	No	Risk-score	200

Author/ Year	miRNA	Country	Study type	N	Assay	Sample	Survival Analysis	Source of HR	Adjusted	Cut-off value	Follow-up time (months)
Shen et al. (2014)[57]	27b-3p	China	Cohort	58	qRT-PCR	FFPE	OS	Report	Yes	ROC curve (3.553)	127
Shen et al. (2014)[57]	27b-3p	China	Cohort	41	qRT-PCR	FFPE	OS	Report	Yes	ROC curve (3.553)	80
Shiet al. (2019)[58]	153	China	Cohort	60	qRT-PCR	Tissue	OS/DSF	Report	Yes	Risk-score	80
Sun et al. (2016) [10]	181a	China	Cohort	47	qRT-PCR	Tissue	OS	Report	Yes	Expression levels	60
Tang et al. (2014)[60]	185	China	Case control	51	qRT-PCR	Tissue	OS/DSF	Report	Yes	Risk-score	120
Toyama et al. (2012)[61]	210	Japan	Cohort	58	qRT-PCR	FFPE	OS	Report	Yes	T/N ratio 4	149
Turashvili et al. (2018)[62]	Let-7d-3p	Canada	Cohort	51	qRT-PCR	FFPE	OS/RFS	Report	No	Quartile	156
Turashvili et al. (2018)[62]	30a-3p	Canada	Cohort	51	qRT-PCR	FFPE	OS/RFS	Report	No	Quartile	156
Turashvili et al. (2018)[62]	30a-5p	Canada	Cohort	51	qRT-PCR	FFPE	OS/RFS	Report	No	Quartile	156
Turashvili et al. (2018)[62]	199a-5p	Canada	Cohort	51	qRT-PCR	FFPE	OS/RFS	Report	No	Quartile	156

Author/ Year	miRNA	Country	Study type	N	Assay	Sample	Survival Analysis	Source of HR	Adjusted	Cut-off value	Follow-up time (months)
Turashvili et al. (2018)[62]	203b-5p	Canada	Cohort	51	qRT-PCR	FFPE	OS/RFS	Report	No	Quartile	156
Turashvili et al. (2018)[62]	324-5p	Canada	Cohort	51	qRT-PCR	FFPE	OS/RFS	Report	No	Quartile	156
Turashvili et al. (2018)[62]	30c-5p	Canada	Cohort	51	qRT-PCR	FFPE	OS/RFS	Report	No	Quartile	156
Uva et al. (2018)[63]	135b	Italy	Cohort	106	qRT-PCR	FFPE	OS	Report	Yes	Risk-score	183
Wang J. et al. (2017)[64]	629-3p	China	Cohort	525	qRT-PCR	FFPE	OS/DFS/DMFS	Report	Yes	Risk-score	200
Wang L. et al. (2019) [65]	205	China	Cohort	40	qRT-PCR	FFPE	OS	SC	No	Median	>90
Wang Q. et al. (2016) [66]	146a	China	Cohort	60	ISH/ qRT-PCR	Tissue	OS	SC	No	Risk-score	98
Wong et al. (2019) [67]	4417	EUA	Cohort	97	qRT-PCR	Tissue	OS	Report	No	Risk-score	60
Yao et al. (2018) [70]	493	China	Cohort	382	ISH	Tissue	OS/DFS	Report	Yes	Median	94,73
Yu et al. (2014)[68]	301a	China	Cohort	118	qRT-PCR	FFPE	OS	Report	Yes	Median	224
Yu et al. (2019)[69]	140-5p	China	Coorte	62	qRT-PCR	Tissue	OS/DFS	SC	No	Expression levels	250
Xiao et al. (2017) [71]	128	China	Coorte	110	qRT-PCR	Tissue	OS/DFS	Report	Yes	Median	110

Author/ Year	miRNA	Country	Study type	N	Assay	Sample	Survival Analysis	Source of HR	Adjusted	Cut-off value	Follow-up time (months)
Zavala et al. (2016)[72]	146a	Chile	Cohort	39	qRT-PCR	FFPE	OS	SC	No	Median	180
Zavala et al. (2016)[72]	638	Chile	Cohort	39	qRT-PCR	FFPE	OS	SC	No	Median	180
Zeng et al. (2017) [73]	34a	China	Cohort	178	qRT-PCR	Blood	OS	Report	No	Risk-score	96
Zeng et al. (2017) [73]	34b	China	Cohort	178	qRT-PCR	Blood	OS	Report	No	Risk-score	96
Zeng et al. (2017) [73]	34c	China	Cohort	178	qRT-PCR	Blood	OS	Report	No	Risk-score	96
Zheng et al. (2018)[74]	301a	China	Cohort	130	ISH	Tissue	OS/DFS	Report	Yes	Median	140

Abbreviations: OS = Overall Survival, DFS = Disease Free Survival, DMFS = Distant Metastasis Free Survival, RFS = Relapse Free Survival, HR = Hazard ratio,, ISH = in situ hybridization, qRT-PCR = quantitative real- time polymerase chain reaction, , IHC= Immunohistochemistry, CISH= In situ Chromogenic Hybridization, NSCA= NanoString nCounter assay, SC= Survival curve, N= Numbers of Subjects, BP= Paraffin Block, FFPE = Formalin fixed paraffin embedded.

Table S2: Descriptive characteristics and statistical data of the studies

		Overall Survival (OS)		Disease-Free Survival (DFS			NOS
Author	miRNAS	HR (95%IC)	*P*	HR (95%IC)	*P*	Expression for poor prognosis	Classification
Cao Z. et al. [35]	361-5p	0.49 (0.28-0.86)	0.012			Low	7
Cao et al. [28]	454	6.40 (1.56-26.32)	0.010	3.81 (1.45-10.00)	0.007	Up	7
Cao et al. [28]	454	6.99 (2.13-22.91)	0.001	3.65 (1.52-08.79)	0.004	UP	7
Cascione et al.[36]	16	0.87 (0.79-0.94)	0.002	0.85 (0.77-0.94)	0.001	Low	7
Cascione et al. [36]	155	0.73 (0.57-0.92)	0.009	0.85 (0.77-0.94)	0.001	Low	7
Cascione et al. [36]	374a	0.85 (0.72-0.99)	0.044	0.79 (0.67-0.93)	0.006	Low	7
Cascione et al. ([36]	125b	1.36 (1.03-1.79)	0.031	1.45 (1.08-1.95)	0.013	Up	7
Cascione et al. ([36]	374b			0.78 (0.66-0.93)	0.005	Low	7
Cascione et al. [36]	497			0.73 (0.55-0.96)	0.024	Up	7
Cascione et al. [36]	655			1.59 (1.02 -2.47)	0.039	Up	7
Cascione et al. ([36]	421			1.25 (1.01 -1.55)	0.042	Up	7
Chen L. et al. [38]	223	5.997(2.18-16.903)	0.001	3.142 (1.063-9.283)	0.038	Low	7
Deng et al. [39]	211-3p	0.0480 (0.263-0.879)	0.017			Low	7
Dong et al. [41]	21	2.32 (1.24-4.12)	0 .033			UP	7
Gasparini et al. [42]	155	0.73 (0.57-0.92)	0 .04			Low	7
Gasparini et al. [42]	493	0.88 (0.72-0.99)	0 .01			Low	7
Gasparini et al. [42]	30e	1.08(1.03-1.79)	0 .04			Low	7
Gasparini et al. [42]	27a	1.09 (1.03-1.79)	0 .01			UP	7

Author	miRNAS	Overall Survival (OS) HR (95%IC)	*P*	Disease-Free Survival (DFS HR (95%IC)	*P*	Expression for poor prognosis	NOS Classification
Gong et al. [43]	378	2.37 (1.18-4.76)	0.016	2.22(1.18-4.18)	0.014	UP	7
Kalniete et al. [45]	214	1.82(1.05-3.14)	0 .0314			UP	7
Gasparini et al. [42]	30e	1.08(1.03-1.79)	0.04			Low	7
Gasparini et al. [42]	27a	1.09 (1.03-1.79)	0.01			UP	7
Gong et al. [43]	378	2.37 (1.18-4.76)	0.016	2.22(1.18-4.18)	0.014	UP	7
Kalniete et al. [45]	214			1.82(1.05-3.14)	0.0314	UP	7
Kalniete et al. [45]	21			1.19(0.18-7.75)	0.628	UP	7
Kalniete et al. [45]	10b			___	0.95	UP	7
Kim et al. [46]			0.0405			Up	6
Kong et al. [47]	155	0.42(0.26-0.68)	< .001			Low	6
Li. HY et al. [29]	301b	1.44	0.047			Up	7
Li. HY et al. [29]	181a-3p	1.02	0.045			Up	7
Li. HY et al. [29]	105	1.60	0.042			Low	7
Li. HY et al. [29]	93-3p	2.16	0.040			Low	7
Liu J. et al. [49]	497	1.49 (1.02-2.18)	0.0391			Low	6
LI. Jie et al. [48]	17-5p	2.56 (1.14-5.76)	0.023			Low	6
Liu. P. et al. [50]	26a	0.39(0.16-0.93)	0.032	0.22(0.03-1.45)	0.115	Low	6
Liu. Y. et al.[51]	27b-3p			2.10(1.17-3.76)	___	Up	7
Liu. Y. et al.[51	126-3p			0.48(0.25-0.91)	___	Low	7
Liu. Y. et al.[51]	218-5p			0.47(0.25-0.87)	___	Low	7
Liu. Y. et al.[51]	374-5p			0.51(0.28-0.92)	___	Low	7

		Overall Survival (OS)		Disease-Free Survival (DFS			NOS
Author	miRNAS	HR (95%IC)	*P*	HR (95%IC)	*P*	Expression for poor prognosis	Classification
LV. Z. et al. [52]	212-5p	0.484(0.228-2.026)	0.049			Low	6
Li. HY et al. [29]	301b	1.44	0.047			Up	7
Li. HY et al. [29]	181a-3p	1.02	0.045			Up	7
Li. HY et al. [29]	105	1.60	0.042			Low	7
Li. HY et al. [29]	93-3p	2.16	0.040			Low	7
Liu J. et al. [49]	497	1.49 (1.02-2.18)	0.0391			Low	6
LI. Jie et al. [48]	17-5p	2.56 (1.14-5.76)	0.023			Low	6
Liu. P. et al. [50]	26a	0.39(0.16-0.93)	0.032	0.22(0.03-1.45)	0.115	Low	6
Liu. Y. et al.[51]	27b-3p			2.10(1.17-3.76)	___	Up	7
Liu. Y. et al.[51	126-3p			0.48(0.25-0.91)	___	Low	7
Liu. Y. et al.[51]	218-5p			0.47(0.25-0.87)	___	Low	7
Liu. Y. et al.[51]	374-5p			0.51(0.28-0.92)	___	Low	7
LV. Z. et al. [52]	212-5p	0.484(0.228-2.026)	0.049			Low	6
Mackenzie et al. [53]	21	3.29 (1.47-7.37)	0.003			Up	6
Radojicic et al. [54]	21	0.85 (0.09-8.29)	>.05	2.49(0.72-8.58)	>.05	Up	7
Radojicic et al. [54]	210	1.97 (0.83-4.65)	0.1220	1.93(0.96-3.89)	0.0658	Up	7
Romero - C. et al [55]	342-3p	-----	0.034			Low	7
Sahlberg et al. [56]	18b	6.65 (1.24-35.57)	0.0268			Up	6
Sahlberg et al. [56]	103	7.14 (1.38-36.95)	0.019			Up	6

		Overall Survival (OS)		Disease-Free Survival (DFS			NOS
Author	miRNAS	HR (95%IC)	*P*	HR (95%IC)	*P*	Expression for poor prognosis	Classification
Sahlberg et al. [56]	107	5.80 (1.22-27.51)	0.027			Up	6
Sahlberg et al. [56]	652	5.35 (1.05-27.20)	0.0433			Up	6
Shen et al.	27b-3p	1.96(1.03-3.72)	0.040			Up	8
Shen et al. [57]	27b-3p	6.69(1.51-29.47)	0.012			Up	8
Schwarzenbacher et al[59]	1287-5p	0.78(0.64-0.96)	0.016			Low	7
Shi et al. [58]	153	0.229(0.064-0.822)	0.024	0.227(0.063-0.814)	0.023	Low	8
Sun et al. [10]	181a	2.36(1.84-3.98)	0.018			Up	7
Tanget al. [60]	185	0.02 (0.00-2.01)	0.097	0.12 (0.01-0.98)	0.048	Low	7
Toyama et al. [61]	210	4.39 (1.00-19.28)	0.049			Up	7
Shen et al. [57]	27b-3p	1.96(1.03-3.72)	0.040			Up	8
Shen et al. [57]	27b-3p	6.69(1.51-29.47)	0.012			Up	8
Schwarzenbacher et al [59]	1287-5p	0.78(0.64-0.96)	0.016			Low	7
Shi et al. [58]	153	0.229(0.064-0.822)	0.024	0.227(0.063-0.814)	0.023	Low	8
Sun et al. [10]	181a	2.36(1.84-3.98)	0.018			Up	7
Tanget al. [60]	185	0.02 (0.00-2.01)	0.097	0.12 (0.01-0.98)	0.048	Low	7
Toyama et al. [61]	210	4.39 (1.00-19.28)	0.049			Up	7
Turashvili et al. [62]	Let-7d-3p	0.106(0.023-0.493)	0.004	0.256(0.088-0.743)	0.013	UP	7
Turashvili et al. [62]	30a-3p	6.529(1.427-29.878)	0.0160	6.054(1.713-21.392)	0.005	Low	7
Turashvili et al.[62]	30a-5p	3.722(1.014-13.661)	0.048	4.076(1.314-12.641)	0.015	Low	7
Turashvili et al.[62]	199a-5p	0.262	0.0282			Low	7
Turashvili et al.[62]	203b-5p	3.927	0.0238			Up	7

Author	miRNAS	Overall Survival (OS) HR (95%IC)	*P*	Disease-Free Survival (DFS HR (95%IC)	*P*	Expression for poor prognosis	NOS Classification
Turashvili et al. [62]	324-5p	3.696	0.0321			Up	7
Turashvili et al.[62]	30c-5p			3.660(1.314-12.641)	0.025	Up	7
Turashvili et al. [62]	95-3p	6.995	0.0337	9.829	0.0072	Up	7
Turashvili et al.[62]	128-3p			0.2276	0.0037	Low	7
Uva et al. [63]	135b	3.26(0.98-10.1)	0.054			Up	9
Wang J. et al. [64]	629-3p	1.3(0.9- 1.8)	0.089			Up	7
Wang L. et al. [65]	205	----	0.011			Low	5
Wang Q. et al. [66]	146a	___	0.021			Low	7
Wong et al. [67]	4417	0.25(0.08-0.76)	0.0086			Low	6
Yao et al. [70]	493			0.51(0.28-0.92)	0.026	Low	7
Yu et al. [68]	301a	2.41(1.29-5.52)	0.023			Up	7
Yu et al. [69]	140-5p	-----	0.031	----	0.04	Low	6
Xiao et al. [71]	128	0.33(0.19-0.59)	< .001			Low	7
Zavala et al.[72]	146a	----	0.0131			Low	6
Zavala et al. [72]	638	----	0.0433			Low	6
Zeng et al. [73]	34a	2.062(1.164-3.654)	0.013			Low	7
Zeng et al. [73]	34b	1.515(0.871-2.638)	0.142			Low	7
Zeng et al. [73]	34c	2.474(1.373-4.460)	0.003			Low	7
Zheng et al. [74]	301a	0.162(0.073-0.361)	0.000	0.193(0.118-0.314)	0.000	Up	7

		Relapse Free Survival (RFS)		Distant metastasis-free survival (DMFS)			NOS
**Author**	**miRNAS**	**HR (95%IC)**	***P***	**HR (95%IC)**	***P***	**Expression for poor prognosis**	**Classification**
Chen et al. [37]	211-5p	2.26 (1.38-4.65)	0.029			Low	7
D. Rinaldis et al. [40]	16-2*			0.55(0.38-0.8)	0.0015	Low	7
D. Rinaldis et al. [40]	766			0.49(0.32-0.77)	0.002	Low	7
D. Rinaldis et al. [40]	381			1.48(1.18-1.87)	9e-04	Low	7
D. Rinaldis et al. [40]	409-5p			1.45(1.17-1.81)	8e-04	Low	7
Jang et al. [44]	9			2.514 (1.106-5.713)	0.028	Up	8
Jang et al [44]	155			2.824 (1.265-6.304)	0.011	Low	8

Abbreviation – NOS (newcastle-ottawa scale)
